# A circulating miRNA signature for early diagnosis of acute kidney injury following acute myocardial infarction

**DOI:** 10.1186/s12967-019-1890-7

**Published:** 2019-04-30

**Authors:** Pei-Chun Fan, Chia-Chun Chen, Chen-Ching Peng, Chih-Hsiang Chang, Chia-Hung Yang, Chi Yang, Lichieh Julie Chu, Yung-Chang Chen, Chih-Wei Yang, Yu-Sun Chang, Pao-Hsien Chu

**Affiliations:** 1 Department of Nephrology, Kidney Research Center, Chang Gung Memorial Hospital, Linkou Medical Center, Chang Gung University College of Medicine, No. 5 Fusing Street, Gueishan Dist., Taoyuan City, 333 Taiwan, ROC; 2 Graduate Institute of Clinical Medical Sciences, Chang Gung University, No. 5 Fusing Street, Gueishan Dist., Taoyuan City, 333 Taiwan, ROC; 3 Molecular Medicine Research Center, Chang Gung University, No 259 Wen-Hwa 1st Road, Kwei-Shan, Taoyuan City, 33302 Taiwan, ROC; 4 Department of Colorectal Surgery, Chang Gung Memorial Hospital, No 259 Wen-Hwa 1st Road, Kwei-Shan, Linkou, Taoyuan City, 33302 Taiwan, ROC; 5 Department of Cardiology, Chang Gung Memorial Hospital, Chang Gung University College of Medicine, No. 5 Fusing Street, Gueishan Dist., Taoyuan City, 333 Taiwan, ROC; 6 Department of Nephrology, Chang Gung Memorial Hospital, Keelung Branch, Chang Gung University College of Medicine, No. 222, Maijin Rd., Anle Dist., Keelung City, 20401 Taiwan, ROC; 7 Department of Cardiology, Chang Gung Memorial Hospital, Chang Gung University College of Medicine, 199 Tung Hwa North Road, Taipei, 105 Taiwan

**Keywords:** MicroRNAs, Acute kidney injury, Acute myocardial infarction, TGF-β

## Abstract

**Background:**

Acute kidney injury (AKI) is a common complication of acute myocardial infarction (AMI), and is associated with adverse outcomes. The study aimed to identify a miRNA signature for the early diagnosis of post-AMI AKI.

**Methods:**

A total of 108 patients admitted to a coronary care unit (CCU) were divided into four subgroups: AMI^−^AKI^−^, AMI^+^AKI^−^, AMI^+^AKI^+^, and AMI^−^AKI^+^. Thirty-six miRNA candidates were selected based on an extensive literature review. Real-time quantitative RT-PCR analysis was used to determine the expression levels of these miRNAs in the serum collected on the day of CCU admittance. TargetScan 7.1 and miRDB databases were used for target prediction and Metacore 6.13 was used for pathway analysis.

**Results:**

Through a stepwise selection based on abundance, hemolytic effect and differential expression between four groups, 9 miRNAs were found to have significantly differential expression levels as potential biomarkers for post-AMI AKI specifically. Noticeably, the expression levels of miR-24, miR-23a and miR-145 were significantly down-regulated in AMI^+^AKI^+^ patients compared to those in AMI^+^AKI^−^ patients. Combination of the three miRNAs as a panel showed the best performance in the early detection of AKI following AMI (AUC = 0.853, sensitivity 95.65%), compared to the analysis of serum neutrophil gelatinase-associated lipocalin (AUC = 0.735, sensitivity 63.16%). Furthermore, bioinformatic analysis indicated that these three miRNAs regulate the transforming growth factor beta signaling pathway and involve in apoptosis and fibrosis in AKI.

**Conclusions:**

For the first time, this study identify a unique circulating miRNA signature (miR-24-3p, miR-23a-3p, miR-145-5p) that can potentially early detect AKI following AMI and may be involved in renal injury and fibrosis in post-AMI AKI pathogenesis.

**Electronic supplementary material:**

The online version of this article (10.1186/s12967-019-1890-7) contains supplementary material, which is available to authorized users.

## Background

Acute kidney injury (AKI) is a common and important complication of various critical illnesses [1]. Among them, the incidence of AKI following acute myocardial infarction (AMI) is approximately 12–37% [2–5]. Compared to those without AKI, patients with AMI who develop incident AKI have significantly prolonged hospital stays and increased rates of hospital mortality (14–39% vs. 0.5–9%) and 1-year mortality (16–50% vs. 5–14%) [2–4, 6, 7]. In the short term, AKI can lead to uremic symptoms, fluid overload, electrolyte imbalance, metabolic acidosis, coagulopathy and increased risk of infection. In addition, the severity and duration of AKI are correlated with the risk of chronic kidney disease and end-stage renal disease, leading to increased economic, social and personal burdens (Additional file 1: Figure S1a) [8, 9].

Although research advances have been made in recent decades, a definite and effective treatment for AKIis still lacking. The best strategies currently focus on prevention, early diagnosis and early interventions aiming at managing the underlying etiologies and complications of AKI. The widely accepted diagnostic criteria for AKI are based on changes of serum creatinine (SCr) and urine output; they include the Risk, Injury, Failure, Loss of kidney function, and End-stage kidney disease (RIFLE) classification [1] the Acute Kidney Injury Network (AKIN) criteria [10] and the Kidney Disease Improving Global Outcomes (KDIGO) criteria [11]. However, obvious changes in SCr may not be seen until 48–72 h after renal insult, potentially delaying the diagnosis of AKI. To enable the early diagnosis of AKI, researchers have investigated various novel biomarkers, such as neutrophil gelatinase-associated lipocalin (NGAL) [12] and kidney injury molecule-1 (KIM-1) [13]. However, these biomarkers may be interfered by diseases other than AKI, and there are controversies regarding cut-offs. To date, researchers have been unable to establish a biomarker-guided clinical strategy that reliably improves the clinical outcome in patients with AKI.

MicroRNAs (miRNAs) are endogenous single-stranded noncoding mRNAs of 19–23 nucleotides that play critical roles in the post-transcriptional regulation of multiple biological cell functions, including proliferation, differentiation, metabolism and apoptosis [14]. Accumulating evidence suggests that certain miRNAs are up- or down-regulated in response to AKI and AMI, respectively. These miRNAs have been suggested as contributing to the pathogeneses of AKI and AMI and as potential biomarkers for both diseases [15, 16]. Nevertheless, no previous study has explored these related miRNA expression levels and functions in post-AMI AKI, and the interaction of miRNAs and their downstream targets also have not been completely elucidated. Furthermore, miRNAs have emerged as promising therapeutic targets in various diseases, including kidney fibrosis and diabetic kidney disease [17], with many ongoing clinical trials. However, the therapeutic implication of miRNAs in AKI has not yet been explored.

This study aims to identify a circulating miRNA signature for post-AMI AKI, in the hopes of facilitating early diagnosis and prompt early intervention. Such miRNAs could also serve as potential therapeutic targets to improve clinical outcomes in patients with post-AMI AKI.

## Methods

### Study design and patient cohort

The study design was summarized in Additional file 1: Figure S1. The study was performed in the coronary care unit (CCU) of a tertiary care referral center. The patients admitted in this CCU from 2010 to 2014 were prospectively enrolled. Patients less than 18 years old, patients who had previously received a kidney transplant, and patients with end-stage renal disease were excluded. The enrolled patients were classified into four groups: AMI^−^AKI^−^, AMI^+^AKI^−^, AMI^+^AKI^+^ and AMI^−^AKI^+^. Demographic characteristics, causes of admission, laboratory data, and hospital outcomes were prospectively collected. The diagnosis of AMI was based on the Consensus Conference criteria for the universal definition of myocardial infarction [18]. AKI was defined based on a change in SCr, as listed in the KDIGO criteria [11]. The study protocol was approved by the local institutional review board, and informed consents were obtained from all participants.

### Sample collection, hemolysis detection, and serum NGAL analysis

Blood samples were collected in non-heparinized tubes within 24 h, at 48 h and at 1 week of admission. Blood samples were centrifuged at 2000 rpm for 10 min, and the obtained serum was stored at − 80 °C for further processing. Serum levels of NGAL were measured in duplicate using commercially available enzyme-linked immunosorbent assay (ELISA) kits according to the manufacturer’s instructions (DLCN20; R&D Systems, Minneapolis, MN, USA). When a measurement exhibited a > 5% variance, a third analysis was performed to ensure a variance of ≤ 5%. For the purpose of early diagnosis, only samples at the first time point (within 24 h) were used to miRNA detection. Before RNA extraction, hemolysis was directly determined in 2 μL serum samples by detecting the absorbance of hemoglobin at 414 nm using the NanoDrop 2000c UV–Vis spectrophotometer (Thermo Scientific, DE, USA).

### Selection of 36 miRNA candidates as potential biomarkers from literature review

A thorough and critical literature review of 119 research articles related to AKI, AMI and cardiovascular diseases (CVD) was performed (Additional file 2: Tables S1, S2). The total reference counts and the numbers of studies related to AKI, AMI and CVD for each miRNA are summarized in Additional file 1: Figure S2a. It is worth noting that many of these miRNAs overlapped in the cardiac and renal diseases. Among the most frequently cited miRNAs, miR-21 was the top one among the 15 miRNAs commonly reported in all three disease types (Additional file 1: Figure S2b). In the 119 studies, miRNAs were detected in various sample types (liquid or tissue biopsies) and species origins (human or murine); the distribution of sample types grouped by disease is shown in Additional file 1: Figure S2c. About 68.2% (30/44) and 66.7% (62/93) of the miRNAs studied in AMI and CVD were tested in patient blood or tissue samples, whereas less studies (27.3%, 24/88) of those studied in AKI were tested in blood, tissue or urine samples. From the compiled data, 36 highly reported miRNAs as the miRNA candidate set were selected.

### RNA extraction and heparinase treatment

An aliquot of 250 μL serum samples were mixed with 1000 μL QIAZol Lysis Reagent (Qiagen) and a spiked-in control consisting of 10^7^ copies of synthetic RNA corresponding to *Caenorhabditis elegans* miR-39 (5′-UCACCGGGUGUAAAUCAGCUUG) (IDT, Coralville, IA, USA). Total RNA was extracted from this mixture using a miRNeasy Mini Kit (Qiagen) according to the manufacturer’s instructions. The purified RNA was eluted in 30 μL nuclease-free water and stored at − 80 °C until use. To avoid the heparin-related interference, 0.5 U heparinase I (Sigma-Aldrich, MO, USA) was added to the pre-reverse transcription (RT) mixture containing 5.4 μL serum RNA, 2 U/μL RNase out (Invitrogen), 1× PCR buffer (Roche Diagnostics) and 1.25 mM MgCl_2_, and the samples were incubated at 25 °C for 1 h.

### Reverse transcription and quantitative polymerase chain reaction

The multiplex RT-qPCR of 37 miRNAs was performed as described previously [19]. The resulting Ct value of each miRNA was first normalized with respect to that of the spiked-in cel-miR-39 by adjusting the miRNA Ct values with the cel-miR-39 ratio (a ratio calculated based on the differences of cel-miR-39 Ct value in each sample to cel-miR-39 median of total samples). To adjust for variations in the total RNA amounts of individual samples, the cel-miR-39-normalized Ct value was further normalized to each individual RNA concentration, which was determined using a NanoDrop 3300 Fluorospectrophotometer (Thermo Scientific, DE, USA) and a Quant-iT™ RiboGreen^®^ RNA Assay (Invitrogen). MiRNAs with normalized Ct values over 40 were defined as ‘undetectable’. Relative miRNA expression levels were represented as 40-Ct.

### miRNA target prediction and pathway analysis

The MiRDB and TargetScan 7.1 databases were used to identify potential human miRNA target genes. The predicted target genes were subjected to Metacore 6.13 pathway map analysis.

### Statistical methods

Continuous variables were summarized as the mean ± standard error and percentages. Normally distributed continuous variables were compared by the Student’s t-test or Fisher’s exact test, and non-normally distributed ones were compared by the Mann–Whitney U test. Categorical variables were compared using the χ^2^ test or Fisher’s exact test. Correlation coefficients were calculated using Spearman’s rank correlation analysis between groups. Non-parametric Kruskal–Wallis test with post hoc Dunn’s tests were conducted to analyze the differences among all groups. The area under the receiver operating characteristic curve (AUC) was used to determine the discriminatory power, and the Youden’s index was used to assess the optimal cut-off value. Algorithms of two- or three-miRNAs panels were built by logistic regression and are, respectively, logit[Pr(Y = 1)] = 13.962 + 0.982 × miR-23a-3p − 1.987 × miR-24-3p and logit[Pr(Y = 1)] = 17.12 + 0.742 × miR-23a-3p − 2.558 × miR-24-3p + 0.772 × miR-145-5p. All statistical tests were 2-tailed, and *P *< 0.05 was considered statistically significant. All statistical analyses were conducted using the IBM SPSS Statistics 22 (SPSS Inc., IL, USA). Plots were graphed using Prism 5 (GraphPad).

## Results

### Patient characteristics

As shown in Table 1, a total of 108 patients were enrolled for analysis and theAMI^−^AKI^−^, AMI^+^AKI^−^, AMI^+^AKI^+^ and AMI^−^AKI^+^subgroups contained 30, 26, 23, and 29 patients, respectively. All four groups have similar gender distribution, body weight, white blood cell count, serum level of alanine aminotransferase, mean arterial pressure, ejection fraction and in-hospital mortality. Compared to AMI^+^AKI^−^ patients, AMI^+^AKI^+^ patients had an older age and higher prevalences of pre-existing hypertension and congestive heart failure. The average SCr values in AMI^+^AKI^+^ and AMI^+^AKI^−^ patients were 3.4 ± 0.5 and 0.9 ± 0.1 mg/dL, respectively. The average serum NGAL levels in AMI^+^AKI^+^ and AMI^+^AKI^−^ patients were 140.1 ± 24.4 and 64.3 ± 7.5 ng/dL, respectively. Compared to AMI^+^AKI^−^ patients, the AMI^+^AKI^+^ patients had lower hemoglobin, lower serum albumin, higher troponin-I and higher B-type natriuretic peptide. Three (13%) of the AMI^+^AKI^+^ patients required renal replacement therapy. Three (13%) of the AMI^+^AKI^+^ patients died during admission.

**Table 1:** Demographic data and clinical characteristics of the study population

Group	AMI^−^AKI^−^ (n = 30)	AMI^+^AKI^−^ (n = 26)	AMI^+^AKI^+^ (n = 23)	AMI^−^AKI^+^ (n = 29)	*P*-value (four groups)	*P*-value (AMI^+^AKI^−^ vs AMI^+^AKI^+^)
Age (years)	61 ± 3	59 ± 2	73 ± 2	71 ± 2	< 0.001	< 0.001
Sex, male (%)	19 (63.3)	22 (84.6)	19 (82.6)	19 (65.5)	0.165	0.576
Body weight (kg)	64.5 ± 1.9	68.9 ± 2.6	65.3 ± 2.3	64.9 ± 2.6	0.563	0.321
Diabetes mellitus (%)	7 (23.3)	7 (26.9)	12 (52.2)	16 (55.2)	0.023	0.064
Hypertension (%)	14 (46.7)	14 (53.8)	20 (87.0)	23 (79.3)	0.004	0.013
Coronary artery disease (%)	8 (26.7)	26 (100.0)	23 (100.0)	8 (27.6)	< 0.001	1.000
Heart failure (%)	13 (43.3)	2 (7.7)	15 (65.2)	22 (75.9)	< 0.001	< 0.001
Blood urea nitrogen (mg/dL)	15 ± 1	14 ± 1	40 ± 6	53 ± 6	< 0.001	< 0.001
Serum creatinine (mg/dL)	0.8 ± 0.1	0.9 ± 0.1	3.4 ± 0.5	3.0 ± 0.3	< 0.001	< 0.001
Serum NGAL (ng/mL)	72.0 ± 9.0	64.3 ± 7.5	140.1 ± 24.4	264.8 ± 56.1	< 0.001	0.007
WBC (10^3^/μL)	8.2 ± 0.4	10.4 ± 0.6	12.6 ± 1.2	11.7 ± 2.0	0.071	0.096
Hemoglobin (g/dL)	13.0 ± 0.4	14.8 ± 0.2	12.2 ± 0.5	11.0 ± 0.5	< 0.001	< 0.001
Sugar (mg/dL)	122 ± 9	171 ± 18	183 ± 20	175 ± 15	0.033	0.671
ALT (U/L)	33 ± 6	31 ± 5	47 ± 11	88 ± 54	0.565	0.222
Albumin (g/L)	3.9 ± 0.1	3.9 ± 0.1	3.4 ± 0.1	3.3 ± 0.1	< 0.001	< 0.001
Troponin I (ng/mL)	2.30 ± 1.13	3.43 ± 1.47	15.98 ± 5.62	0.83 ± 0.27	0.005	0.041
BNP (pg/mL)	386 ± 112	243 ± 132	1098 ± 303	1460 ± 288	0.001	0.038
Mean arterial pressure (mmHg)	87 ± 3	89 ± 3	90 ± 3	86 ± 4	0.759	0.804
Ejection fraction (%)	55 ± 3	58 ± 3	48 ± 4	50 ± 4	0.226	0.050
Renal replacement therapy	0 (0)	0 (0)	3 (13.0)	4 (13.8)	0.021	0.096
In-hospital mortality	1 (3.3)	0 (0)	3 (13.0)	2 (6.9)	0.237	0.096

Values are mean ± standard error

*AMI* acute myocardial infarction, *AKI* acute kidney injury, *ALT* alanine aminotransferase, *BNP* B-type natriuretic peptide, *BUN* blood urea nitrogen, *NGAL* neutrophil gelatinase-associated lipocalin, *RRT* renal replacement therapy, *WBC* white blood cell

### miR-24, miR-23a and miR-145 are the most promising targets for the early detection of post-AMI AKI

**Fig. 1:**
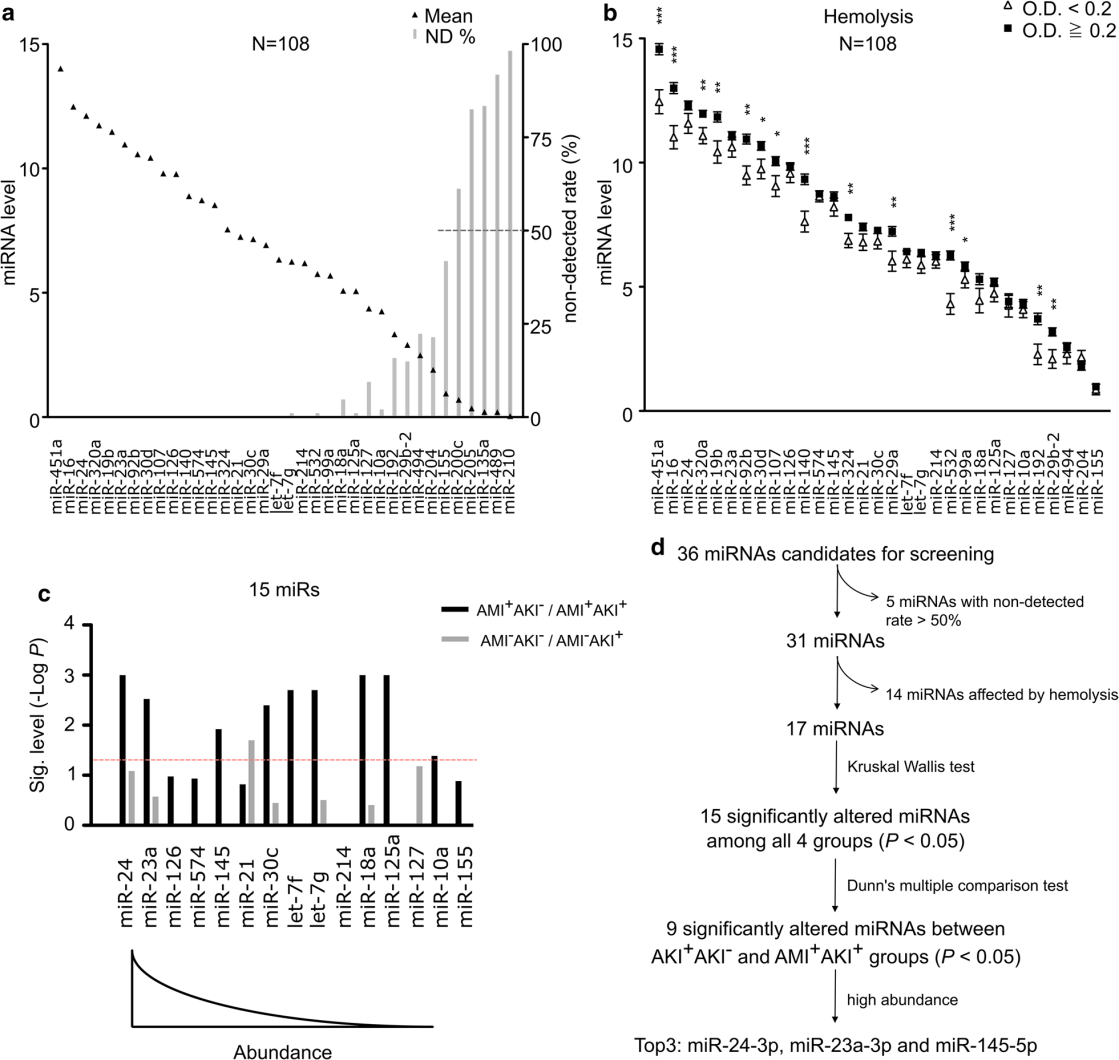
Strategy for identifying circulating miRNAs with the best discriminatory power in detecting post-AMI AKI. **a** Quantification of 36 miRNAs by qRT-PCR in 108 serum samples. The means of individual miRNA expressions are presented as 40-Ct, and sorted in a descending order. The gray bars indicate the non-detected rate (%) of each miRNA, which was defined as the percentage of samples with Ct > 40. **b** Sample hemolysis was determined by the absorbance of hemoglobin at 414 nm greater than 0.2. The means and standard errors for the expression levels of 31 miRNAs in the hemolytic and the non-hemolytic groups were calculated. (*P < 0.05; **P < 0.01; ***P < 0.001). **c** 15 of 17 miRNAs are significantly altered among all 4 groups (determined by non-parametric Kruskal–Wallis test). The significant levels of the 15 miRNAs of the AMI^+^AKI^−^ versus AMI^+^AKI^+^ groups and the AMI^−^AKI^−^ versus AMI^−^AKI^+^ groups are indicated (− Log *P*-values above 1.301 was considered significant). *P*-values are calculated by Dunn’s multiple comparison test. **d** Flow chart displaying the step-wise strategy for practical miRNA biomarkers selection. *AKI* acute kidney injury, *AMI* acute myocardial infarction, *AUC* area under receiver operating characteristic curve, *ND* not detected

A work flow diagram for our identification of the clinically applicable miRNAs with the best discriminatory power in detecting post-AMI AKI is shown in Fig. 1. To increase data reliability, the five least abundant miRNAs (miR-200c, miR-205, miR-135a, miR-489 and miR-210), which were undetectable (Ct value > 40) in more than 50% of our samples, were eliminated (Fig. 1a). In serum samples, hemolysis may cause miRNAs to be released from red blood cells, substantially altering the circulating miRNA content [20]. To exclude the miRNAs whose levels may be altered by hemolysis, the serum samples were separated into non-hemolytic (n = 28) and hemolytic (n = 80) groups by their OD_414 nm_ values of hemoglobin, and then compared miRNA expression between the groups. As shown in Fig. 1b, the expression levels of 14 of 31 miRNAs were significantly increased in hemolytic samples. Among them, miR-451a showed that largest alteration, with a 4.3-fold change in hemolytic samples; this finding is consistent with many previous reports [21–23]. Finally, the remaining 17 miRNA candidates were compared among all 4 groups by Kruskal–Wallis test. Among them, 15 miRNAs were significantly altered between these four groups (Additional file 1: Table S3). Noticeably, after multiple comparisons, 9 of them had significantly differential expression in AMI patients who developed AKI compared to those without developed AKI (Fig. 1c and Additional file 2: Table S1). However, these miRNAs were not altered between AMI^−^AKI^+^ and AMI^−^AKI^−^ group and these data disclosed them specifically as potential biomarkers for post-AMI AKI. In order to facilitate clinical practice with minimum miRNA targets, the top 3 highly abundant miRNAs (miR-24, miR-23a and miR-145)were selected as the most potential biomarkers for further evaluating (Fig. 1c, d).

**Fig. 2:**
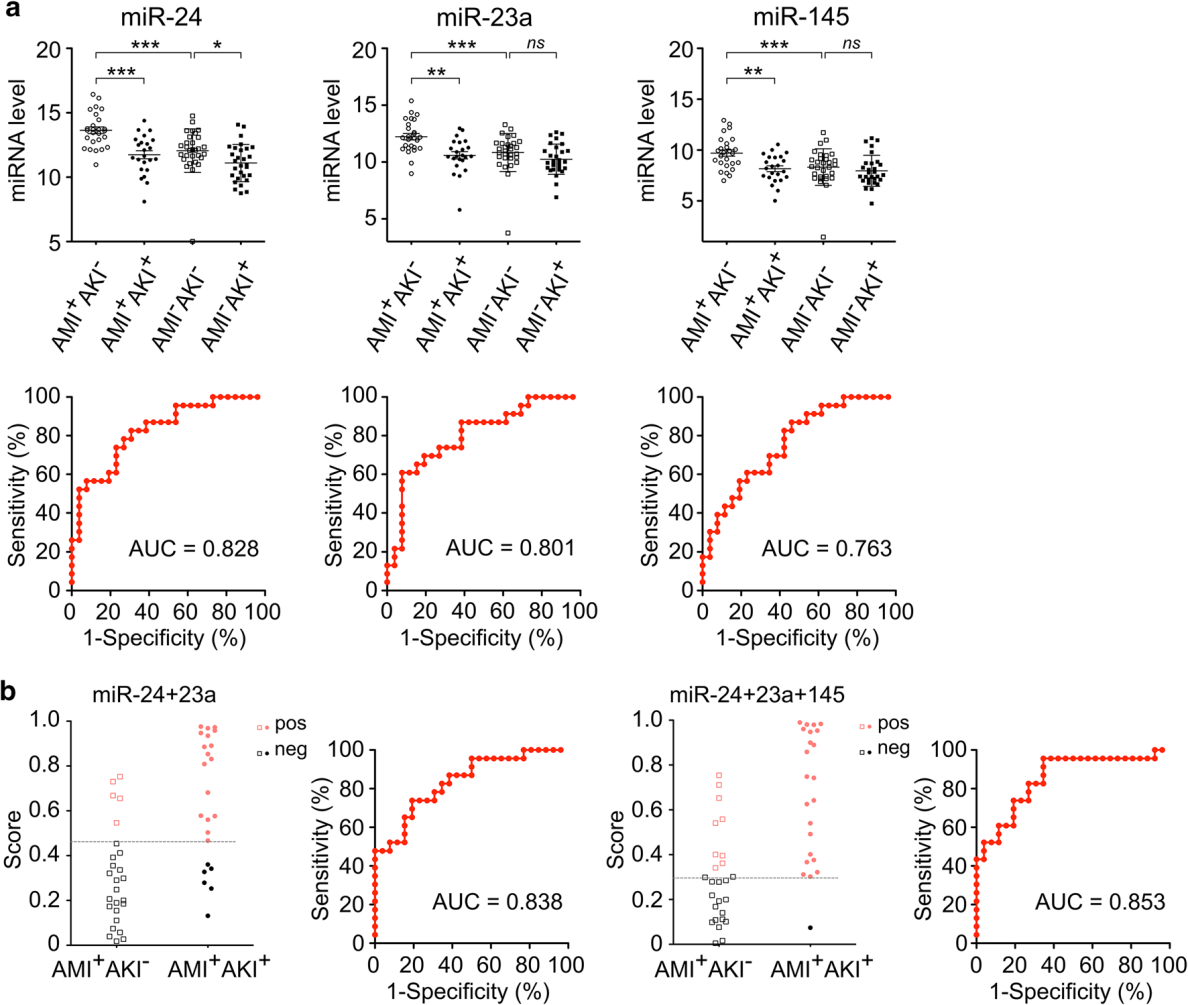
The expression levels and diagnostic performances of miR-24, miR-23a and miR-145 in detecting post-AMI AKI. **a** Scatter plots representing the distributions for the expression levels of, miR-24, miR-23a and miR-145 in the AMI^+^AKI^−^ group (open squares) and the AMI^+^AKI^+^ group (full circles). *P*-value was calculated by Mann–Whitney U test. (*P < 0.05; **P < 0.01; ***P < 0.001; *ns* not significant). The ROC analysis and the AUC values were performed to discriminating between the AMI^+^AKI^−^ and the AMI^+^AKI^+^ groups. **b** Scatter plots presenting the distributions of scores generated by logistic regression integrating the combined effects of miR-24 + miR-23a, or miR-24 + miR-23a + miR-145, in discriminating between the AMI^+^AKI^−^ and AMI^+^AKI^+^ groups. Scores ranging from 0 to 1 were generated for each sample and used to calculate ROC curves. The positive cases (red dots) in each group were determined according to the individual cut-off value obtained using Youden’s index on the ROC curve. *AKI* acute kidney injury, *AMI* acute myocardial infarction, *AUC* area under receiver operating characteristic curve, *ROC* receiver-operating characteristic

The expression levels of miR-24, miR-23a and miR-145were significantly down-regulated in AMI^+^AKI^+^ patients compared to AMI^+^AKI^−^ patients (Fig. 2a; 0.27-, 0.32- and0.34-fold, respectively). For discrimination ability, the AUC values of miR-24 and miR-23a were greater than 0.8, and all three miRNAs had better performances than that of serum NGAL (AUC = 0.735). Using the cut-off values obtained from Youden’s index on the ROC curves of miRNAs and serum NGAL, sensitivities ranging from 60.87 to 86.96% and specificities of 53.85–92.31% were obtained (Table 2). The sensitivity of miR-23a was very close to that of serum NGAL; however, the sensitivities of miR-24 and miR-145 were greatly improved (by ~ 20%). These results strongly suggestedt hat the selected miRNAs may have potential for clinical application.

**Table 2:** Summarized diagnostic factors of the individual miRNAs, combined miRNA panels and serum NGAL for AKI

Variate	Value (mean ± SE)		Fold-change (AMI^+^AKI^+^/AMI^+^AKI^−^)	*P* (MWU test)	Cut-off	AUC (95% CI)	Sensitivity (%)	Specificity (%)
	AMI^+^AKI^−^	AMI^+^AKI^+^						
miR-24 (Ct)	13.64 ± 0.26	11.74 ± 0.31	0.27	< 0.001	< 12.99	0.828 (0.711–0.941)	82.61	69.23
miR-23a (Ct)	12.23 ± 0.28	10.59 ± 0.32	0.32	< 0.001	< 10.89	0.801 (0.676–0.927)	60.87	92.31
miR-145 (Ct)	9.70 ± 0.31	8.16 ± 0.29	0.34	0.002	< 9.67	0.763 (0.631–0.894)	86.96	53.85
sNGAL (ng/mL)	64.32 ± 7.50	140.13 ± 24.38	2.18	0.008	> 87.45	0.735 (0.578–0.892)	63.16	84.62
miR-24 + miR-23a (score)	0.30 ± 0.04	0.66 ± 0.06	2.16	< 0.001	> 0.46	0.838 (0.728–0.948)	73.91	80.77
miR-24 + miR-145 (score)	0.29 ± 0.04	0.67 ± 0.06	2.27	< 0.001	> 0.54	0.843 (0.730–0.956)	69.57	88.46
miR-23a + miR-145 (score)	0.35 ± 0.04	0.61 ± 0.05	1.74	< 0.001	> 0.58	0.801 (0.676–0.927)	60.87	92.31
miR-24 + miR-23a + miR-145 (score)	0.29 ± 0.04	0.67 ± 0.06	2.29	< 0.001	> 0.30	0.853 (0.744–0.962)	95.65	65.38

*AKI* cute kidney injury, *AMI* acute myocardial infarction, *AUC* area under receiver operating characteristic curve, *CI* confidence interval, *Ct* cycle threshold, *MWU* Mann–Whitney U, *NGAL* neutrophil gelatinase-associated lipocalin, *SE* standard error

### The miRNA panel (miR-24, miR-23a, miR-145) has the highest detection rate of post-AMI AKI

Based on the high sensitivity of miR-24 and miR-145 and the high specificity of miR-23a, the top two miRNAs (miR-24 and miR-23a) and the top three miRNAs (miR-24, miR-23a and miR-145) were combined into panels, and used logistic regression analysis to evaluate the performance of these panels in detecting post-AMI AKI. As shown in Fig. 2b, the scores of the two-miRNA model and three-miRNA model were elevated in the AMI^+^AKI^+^ group compared with those in the AMI^+^AKI^−^ group. Using the two-miRNApanel with the cut-off value of 0.46 yielded a slightly higher AUC value of 0.838, a sensitivity of 73.91% and a specificity of 80.77%. Using the three-miRNA panel with the cut-off value of 0.30 yielded an AUC value of 0.853, a remarkably increased sensitivity of 95.65% and a moderate specificity of 65.38% (Table 2). This three-miRNA signature detected 22 out of the 23 patients in the AMI^+^AKI^+^ group and had the best performance for diagnosing post-AMI AKI.

### miR-24, miR-23a and miR-145 are simultaneously up-regulated in AMI, but down-regulated in AKI

Our literature review indicated that miR-24, miR-23a and miR-145 were highly related to AKI and AMI (Additional file 1: Figure S2), and our experimental results showed that all three miRNAs were significantly downregulated in AMI^+^AKI^+^ patients compared to those in AMI^+^AKI^−^ patients (Fig. 2a). miRNAs in the AMI^−^AKI^+^and AMI^+^AKI^−^ groups were also examined. Comparing to the AMI^−^AKI^−^ group (disease control), the AMI^+^AKI^−^ group exhibited significantly higher expression levels of miR-24 (threefold, *P *< 0.001), miR-23a (2.62-fold, *P *= 0.001) and miR-145 (2.6-fold, *P *= 0.004). In contrast, AMI^−^AKI^+^samples exhibited decreased expression levels of miR-24 (0.52-fold, *P *= 0.004), miR-23a (0.67-fold, *P *= 0.024) and miR-145 (0.78-fold, *P *= 0.098). These data may explain the dramatic down-regulation of these three miRNAs in AMI patients upon the development of AKI, and could suggest that theses miRNAs play relevant roles in AMI and AKI.

### miR-24, miR-23a and miR-145 all target on TGF-β signaling and apoptosis pathway

**Fig. 3:**
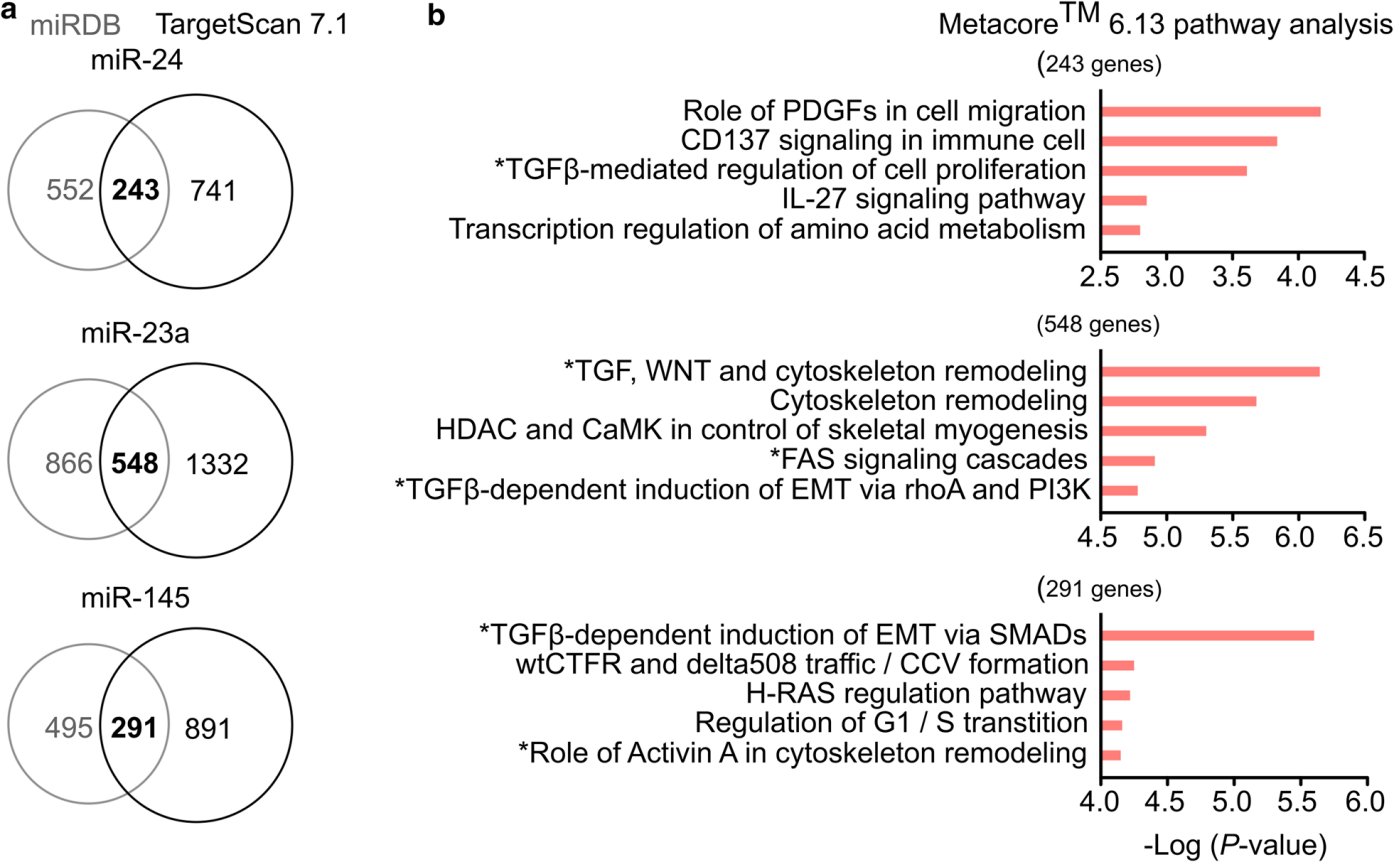
Target prediction and pathway analysis of miR-24, miR-23a and miR-145 target genes. **a** Venn diagrams showing the potential miRNA targets predicted from TargetScan 7.1 and MiRDB. **b** The lists of intersecting genes were further examined by Metacore™ 6.13 pathway map analysis. The top five significantly enriched pathways are listed with their − Log *P*-values. TGF-β- or apoptosis-associated pathways are marked with asterisks (*). *TGF*-*β* transforming growth factor beta

In order to demonstrate the possible functions involved in post-AMI AKI, our miRNA target analysis by MiRDB and TargetScan 7.1 databases identified 243, 548 and 291 predicted targets of miR-24, miR-23a and miR-145, respectively (Fig. 3a). Through Metacore pathway map analysis, the top five significantly enriched pathways for each miRNAare shown in Fig. 3b. Impressively, all of the three miRNAs are involved in two common pathways, TGF-β signaling and apoptosis, in kidney injury. Among the 32 predicted target genes related to TGF-β signaling and apoptosis pathway, 14 target genes (ACVR1B, APAF1, CASP7, FAS, LRP5, MAPK14, PDGFRB, PXN, SERPINE1/PAI1, SMAD2, SMAD3, TCF3, TGFBR2 and XIAP) had been experimentally proved by previous studies (Table 3). All these data support that the three miRNAs simultaneously regulate TGF-β signaling and apoptosis pathway.

**Table 3:** Predicted and experimentally validated target genes of miR-24, miR-23a and miR-145 in TGFβ- and apoptosis-related pathways

Target		miRNA	
Gene symbol	Pathway	Prediction	Experiment
ACVR1B	Role of activin A in cytoskeleton remodeling	miR-145	miR-24 (2), miR-145 (6,11)
ACVR2A	Role of activin A in cytoskeleton remodeling	miR-145	
APAF1	FAS signaling cascades	miR-23a	miR-23a (17,20,23,25,28,34,42)
CASP7	FAS signaling cascades	miR-23a	miR-23a (7,12,21)
CFL2	TGF, WNT and cytoskeleton remodeling/TGFβ-dependent induction of EMT via rhoA and PI3 K	miR-23a	
CHUK	TGF, WNT and cytoskeleton remodeling/TGFβ-dependent induction of EMT via rhoA and PI3K	miR-23a	
COL4A1	TGF, WNT and cytoskeleton remodeling	miR-23a	
FAS	FAS signaling cascades	miR-23a	miR-23a (3,15,22)
GSK3B	TGF, WNT and cytoskeleton remodeling/TGFβ-dependent induction of EMT via rhoA and PI3K	miR-23a	
LRP5	TGF, WNT and cytoskeleton remodeling	miR-23a	miR-23a (36), miR-145 (38)
MAP3K1	FAS signaling cascades	miR-23a	
MAP3K5	FAS signaling cascades	miR-23a	
MAPK14	TGFβ-mediated regulation of cell proliferation	miR-24	miR-24 (1)
MKL2	TGFβ-dependent induction of EMT via SMADs	miR-145	
MYL12B	TGF, WNT and cytoskeleton remodeling	miR-23a	
NLK	TGF, WNT and cytoskeleton remodeling	miR-23a	
PAK2	FAS signaling cascades	miR-23a	
PDGFRA	TGFβ-mediated regulation of cell proliferation	miR-24	
PDGFRB	TGFβ-mediated regulation of cell proliferation	miR-24	miR-24 (16,33)
PDPK1	TGFβ-dependent induction of EMT via rhoA and PI3 K	miR-23a	
PIK3C2A	TGF, WNT and cytoskeleton remodeling/TGFβ-dependent induction of EMT via rhoA and PI3K	miR-23a	
PIK3CB	TGF, WNT and cytoskeleton remodeling/TGFβ-dependent induction of EMT via rhoA and PI3K	miR-23a	
PPP1CB	TGF, WNT and cytoskeleton remodeling	miR-23a	
PPP1R12A	TGF, WNT and cytoskeleton remodeling	miR-23a	
PXN	Role of activin A in cytoskeleton remodeling	miR-145	miR-145 (30)
SERPINE1/PAI1	TGFβ-dependent induction of EMT via SMADs	miR-145	miR-145 (8,13)
SMAD2	TGFβ-dependent induction of EMT via SMADs/role of activin A in cytoskeleton remodeling	miR-145	miR-145 (5,40)
SMAD3	TGFβ-dependent induction of EMT via SMADs/role of activin A in cytoskeleton remodeling	miR-145	miR-23a (39,41), miR-145 (14,27,29,31,37)
TCF3	TGFβ-dependent induction of EMT via SMADs	miR-145	miR-24 (19)
TGFBR2	TGF, WNT and cytoskeleton remodeling/TGFβ-dependent induction of EMT via rhoA and PI3K/TGFβ-dependent induction of EMT via SMADs	miR-23a, miR-145	miR-145 (18,26,40)
TJP1	TGFβ-dependent induction of EMT via rhoA and PI3K	miR-23a	
XIAP	TGF, WNT and cytoskeleton remodeling/FAS signaling cascades	miR-23a	miR-23a (4,9,35), miR-24 (10,24,32)

The references are listed in Additional file 2: Supplementary references

## Discussion

In the present study, a thorough literature review was performed to select 36 miRNA candidates as potential biomarkers for post-AMI AKI. From among them, serum miR-24, miR-23a and miR-145 were found to be significantly down-regulated in AMI^+^AKI^+^ patients and showed good discriminatory power (i.e., better than serum NGAL) in detecting in post-AMI AKI within 24 h of admission. Our logistic regression analysis showed that this three-miRNA panel exhibited better diagnostic performance than the individual miRNAs or a two-miRNA panel. Our target prediction and pathway analyses showed that these three miRNAs commonly regulate TGF-β signaling and apoptosis. To our knowledge, this is the first report to identify a unique circulating miRNA signature for detecting post-AMI AKI at an early stage and demonstrate their pathogenic mechanisms.

Our results demonstrated that the three-miRNA panel had excellent performance in the early the diagnosis of post-AMI AKI. Over the last decade, NGAL has been known as one of the most promising and sensitive novel protein biomarkers for diagnosing AKI, with a reported AUC of 0.815 [24]. When NGAL was combined with KIM-1 and *N*-acetyl-beta-d-glucosaminidase (NAG), the AUC of the three-protein panel could reach 0.840 [25]. In our current study, using samples obtained within 24 h of admission, miR-24 alone (AUC = 0.828), miR-23a alone (AUC = 0.801), miR-145 alone (AUC = 0.763) and the three-miRNA panel (AUC = 0.853) all had higher discriminatory abilities than serum NGAL (AUC = 0.735) in diagnosing post-AMI AKI. Our data indicate that the three-miRNA panel is a promising diagnostic tool for post-AMI AKI.

To determine this signature for post-AMI AKI, three disease control groups were used from CCU patients (AMI^−^AKI^−^, AMI^+^AKI^−^and AMI^−^AKI^+^) rather than normal healthy individuals. Previous studies separately discussed miRNA expression under AMI or AKI; however, both conditions have shared pathophysiologies including cell apoptosis, inflammation, and fibrosis. Some miRNAs were reported to be involved in both AMI and AKI, but it was not known whether they acted synergistically or antagonistically. The use of disease control groups allowed us to focus on the specific effect of AKI on miRNA expression among AMI patients, in turn enabling us to identify a circulating miRNA signature that is distinctive in diagnosing post-AMI AKI.

MiR-24, miR-23a and miR-145 had all been previously reported in AMI, and miR-24 and miR-145 had been implicated in AKI. MiR-24 was reportedly down-regulated in the plasma of critically ill patients with AKI [26]. Its tissue levels were up-regulated in a mouse model of renal ischemic reperfusion (I/R) injury and in renal transplant patients with prolonged cold ischemic time [27]. MiR-24 was shown to be induced by hypoxia inducible factor-1, and to thereby promote renal ischemic injury by stimulating apoptosis in endothelial and tubular epithelial cells. In vivo inhibition of miR-24 was shown to reduce tubular injury and improve renal function [27]. MiR-24 was found to be specifically enriched in cardiac endothelial cells in the infarct border zone after AMI [28]. Inhibition of endothelial miR-24 limited the myocardial infarct size of mice by preventing endothelial apoptosis and enhancing vascularity, thereby preserving cardiac function and survival. MiR-23a was found to be up-regulated in the plasma of patients with coronary artery diseases, and in an I/R-induced myocardial infarction model [29, 30]. MiR-23a was also shown to regulate cardiomyocyte apoptosis by inhibiting manganese superoxide dismutase [31]. MiR-145 is reportedly abundant in normal vascular smooth muscle cells, but down-regulated in injured or atherosclerotic vessels [32]. In contrast to patients with coronary artery diseases, the plasma levels of miR-145 were found to be increased in AMI patients; moreover, these levels were positively correlated with the infarct size [33]. Our current results agree with these previous reports and demonstrate for the first time that miR-23a is down-regulated in AKI. The distinctive expression pattern of these three miRNAs suggests that they may be functionally relevant.

**Fig. 4:**
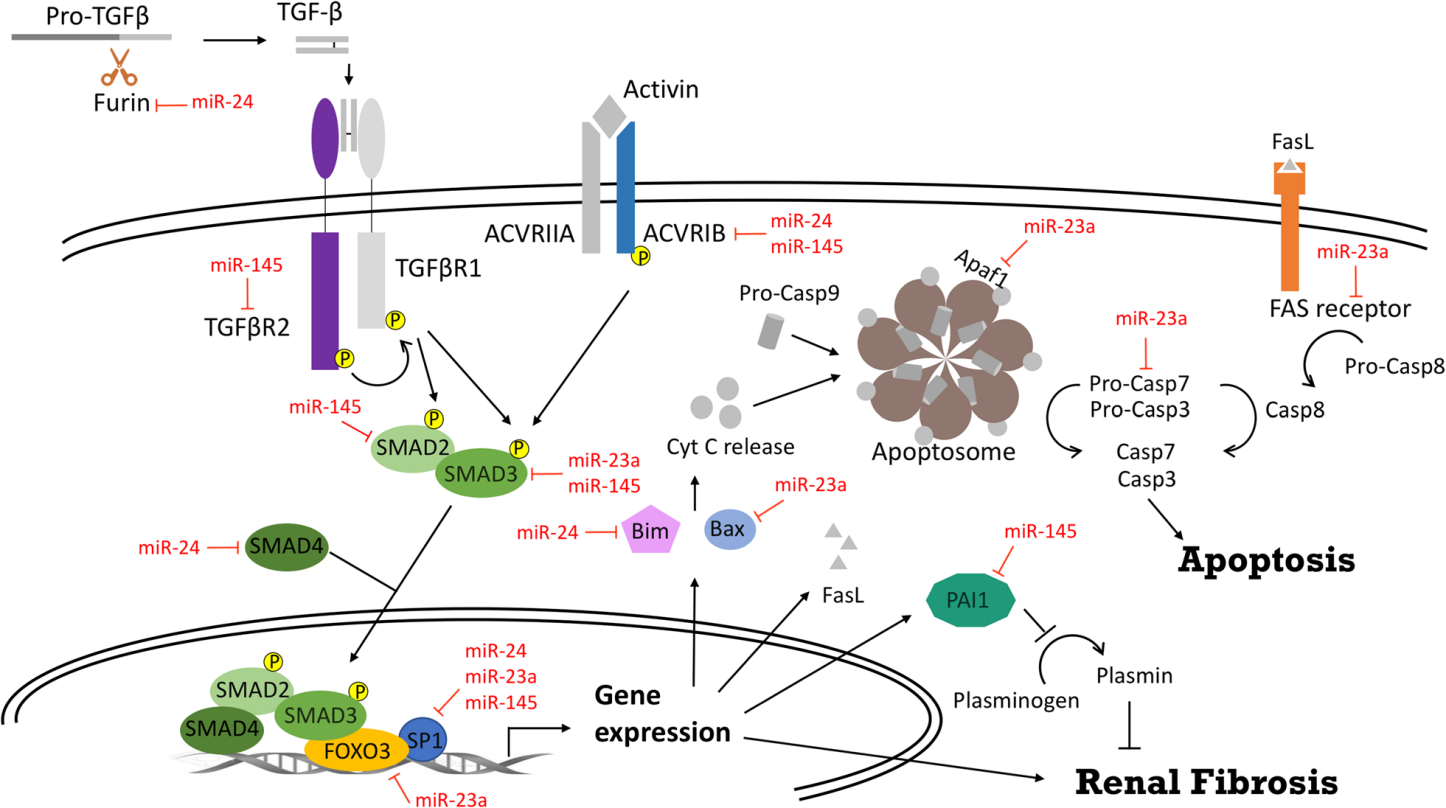
Schematic overview for the roles of miR-24, miR-23a and miR-145 in TGF-β signaling in AKI. The proteolytic activity of Furin is required for the maturation of TGF-β. After binding with mature TGF-β, TGFβR2 phosphorylates TGFβR1 and induces canonical SMAD2/3 signaling. Phosphorylated SMAD2/3 interacts with SMAD4, and this complex translocates into the nucleus and associates with transcription factors (FOXO3 and SP1) to regulate target gene expression. The pro-apoptotic factors, Bim and Bax, are induced by TGF-β-SMADs signaling to cooperatively trigger the release of Cyt C from mitochondria. Cyt C, Pro-Casp9 and Apaf1 form the apoptosome, which can initiate intrinsic apoptosis through the caspase 9-mediated cleavage of caspase 3/7. FasL is transactivated by the canonical TGF-β pathway, binds to Fas receptor, and enhances extrinsic apoptosis by autolysis of Pro-Casp8. Activated caspase 8 also converts caspase 3/7 into active enzymes and induces cell death. SMAD2/3 transactivates pro-fibrotic genes that are directly involved in extracellular matrix deposition. PAI1 is also activated via the TGF-β-SMADs pathway; activated PAI1 reduces the production of plasmin and protects fibrin from degradation, thereby promoting tissue fibrosis. The experimentally confirmed targets of miR-24, miR-23a and miR-145 are marked. *AKI* acute kidney injury, *Apaf1* apoptotic protease activating factor 1, *Cyt C* cytochrome C, *FasL* Fas ligand, *FOXO3* forkhead box O3, *PAI1* plasminogen activator inhibitor-1, *Pro*-*Casp8* pro-caspase 8, *Pro*-*Casp9* pro-caspase 9, *TGF*-*β* transforming growth factor beta, *TGFBR1* transforming growth factor, beta receptor I, *TGFBR2* transforming growth factor, beta receptor II, *SP1* specificity protein 1

Our bioinformatic study found that miR-24, miR-23a and miR-145 were commonly associated with TGF-β signaling and apoptosis. TGF-β is well recognized as a key mediator of renal fibrosis [34]. Evidence also showed that TGF-β signaling participates in kidney injury. During AKI, tubular epithelial cells can produce TGF-β and fractalkines to promote inflammation and kidney injury [35]. Deletion the TGF-β type II receptor in the proximal tubules could attenuate tubular apoptosis and reduce renal injury in a mouse model, suggesting that TGF-β signaling is pro-apoptotic in this setting [36]. In a transgenic mouse model, activation of TGF-β signaling in the tubular epithelium alone was sufficient to cause AKI [37]. Tubular cell apoptosis, which is well recognized as the hallmark of AKI, involves intrinsic, extrinsic and endoplasmic reticulum stress pathways [38]. Previous studies showed that the three miRNAs selected in the present work can target TGF-β signaling and apoptosis by regulating Furin, SMAD4, Bim, Bax, FOXO3, and SP1. Based on the bioinformatic analysis and previous experimental studies, a network model was proposed (Fig. 4) in which the three selected miRNAs suppress the expressions of TGF-β signaling components (Furin, TGFBR2 and SMADs), the transcription factors (Foxo3 and SP1), and apoptosis-related molecules (Bim, Bax, Caspase 7, Apaf-1 and Fas receptor). Taken together, the three selected miRNAs not only have the potential to serve as early biomarkers for detecting post-AMI AKI, but may also contribute to post-AMI AKI pathogenesis by regulating TGF-β signaling and apoptosis to promote renal injury and fibrosis. The biological relevance and therapeutic potential of these three miRNAs in AKIare worthy of further study.

## Limitations

The study population was relatively small and not enough to evaluate the correlation of the three selected miRNAs with the severity of AKI or the long-term clinical outcome. Further investigation is needed to validate the accuracy and clinical utility of the three-miRNA panel to serve as an early detection tool of AKI following AMI or other cardiorenal syndromes.

## Conclusion

In conclusion, for the first time, this study identified a unique circulating miRNA signature (miR-24, miR-23a and miR-145) that enables the early detection of post-AMI AKI in human. These three miRNAs target TGF-β signaling and apoptosis, and may be involved in renal injury and fibrosis in post-AMI AKI pathogenesis.

## Additional files

**Additional file 1: Figure S1.** Study design. **Figure S2.** Summary of total reference numbers, disease distribution and sample type analysis of the miRNA candidates. **Table S3.** Kruskal–Wallis test and post hoc Dunn’s multiple comparisons of the 17 miRNAs among all groups (N = 108).

**Additional file 2: Tables S1, S2.** Literature review of 119 research articles related to AKI, AMI and cardiovascular diseases. **Supplementary references.** Supplementary references of Additional file 1: Table S3.

## Abbreviations

ACVR1B: activin A receptor type 1B
AKI: acute kidney injury
AKIN: Acute Kidney Injury Network
AMI: acute myocardial infarction
APAF1: apoptotic protease activating factor 1
AUC: area under receiver operating characteristic curve
CASP7: caspase 7
CCU: coronary care unit
CKD: chronic kidney disease
CVD: cardiovascular disease
ELISA: enzyme-linked immunosorbent assay
FasL: Fas ligand
FOXO3: forkhead box O3
I/R: ischemic reperfusion
KDIGO: Kidney Disease Improving Global Outcomes
KIM-1: kidney injury molecule-1
LRP5: low-density lipoprotein-related receptors 5
MAPK14: mitogen-activated protein kinase 14
miRNA: microRNA
NAG: *N*-acetyl-beta-d-glucosaminidase
ND: not detected
NGAL: neutrophil gelatinase-associated lipocalin
PAI1: plasminogen activator inhibitor-1
PCR: polymerase chain reaction
PDGFRB: platelet-derived growth factor receptor beta
Pro-Casp8: pro-caspase 8
Pro-Casp9: pro-caspase 9
PXN: paxillin
RIFLE: Risk, Injury, Failure, Loss of kidney function, and End-stage kidney disease
ROC: receiver-operating characteristic
SCr: serum creatinine
SERPINE1: serpin family E member 1
SP1: specificity protein 1
TCF3: transcription factor 3
TGF-β: transforming growth factor beta
TGFBR1: transforming growth factor, beta receptor I
TGFBR2: transforming growth factor, beta receptor II
XIAP: X-linked inhibitor of apoptosis protein
