# Supplementary material for: A circulating miRNA signature for early diagnosis of acute kidney injury following acute myocardial infarction
Source: J Transl Med. 2019 Apr 30;17:139. doi: 10.1186/s12967-019-1890-7 (PMC6492315; doi:10.1186/s12967-019-1890-7)
Supplement: Supplementary file 1 — Additional file 1: Figure S1. Study design. Figure S2. Summary of total reference numbers, disease distribution and sample type analysis of the miRNA candidates. Table S3. Kruskal–Wallis test and post hoc Dunn’s multiple comparisons of the 17 miRNAs among all groups (N = 108). [file 12967_2019_1890_MOESM1_ESM.docx]

**Figure S1** Study design.


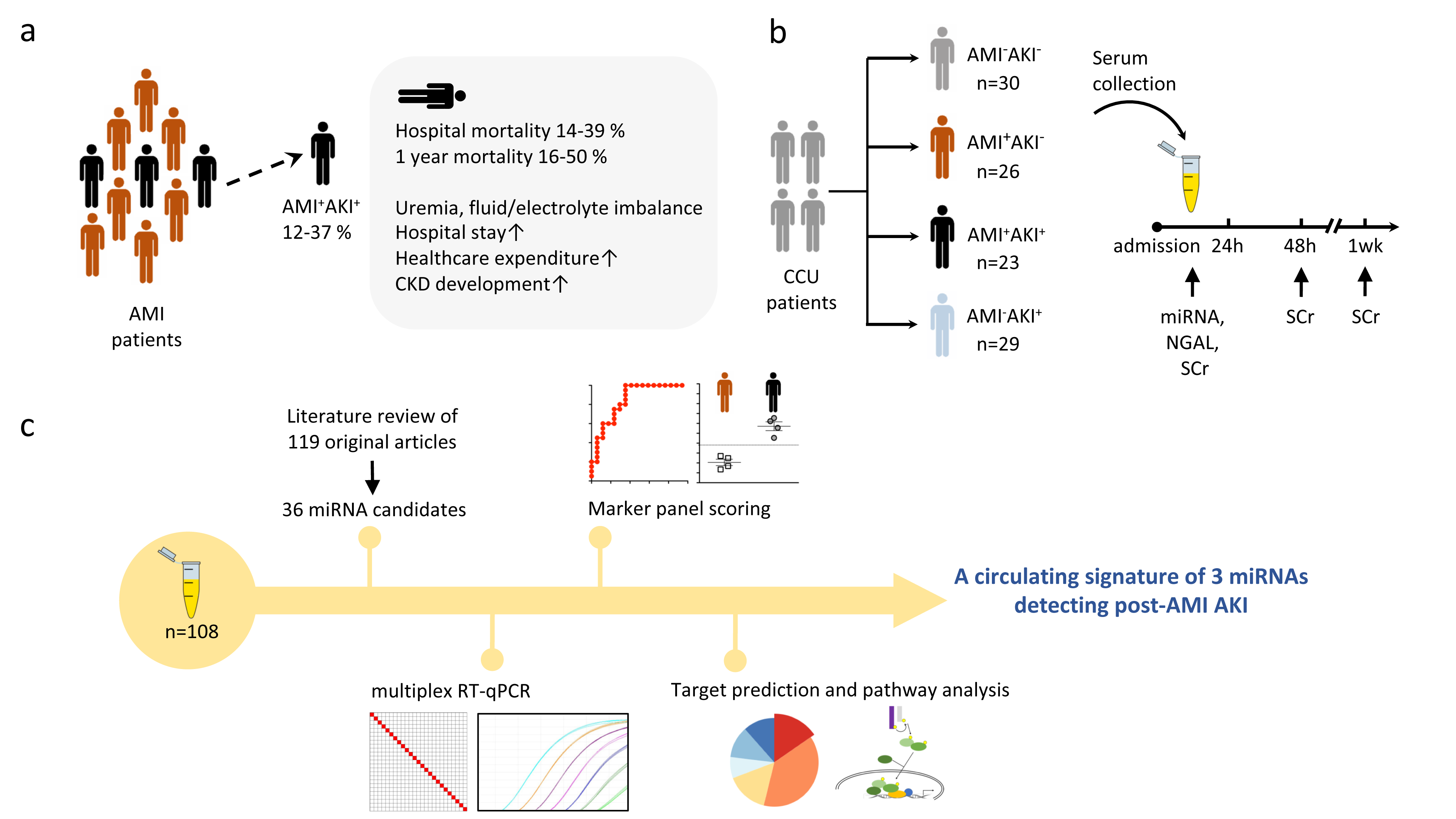


**a** The incidence of AKI among AMI patients is 12-37%. Post-AMI AKI is associated with increased mortality, uremia, fluid/electrolyte imbalance, increased hospital stays, increased healthcare expenditure and chronic kidney disease development. **b** A total of 108 enrolled CCU patients were divided into four groups: AMI^-^AKI^-^, AMI^+^AKI^-^, AMI^+^AKI^+^ and AMI^-^AKI^+^. Serum samples were collected for miRNA/NGAL/SCr analysis. **c** Thirty-six miRNA candidates were selected through a literature review. Their expression levels in the 108 serum samples were detected by multiplex RT-qPCR. A marker panel was determined by statistical analysis. Finally, target prediction and pathway analysis were conducted to study the biological relevance of the miRNA panel. *AKI* acute kidney injury, *AMI* acute myocardial infarction, *CCU* coronary care unit, *CKD* chronic kidney disease, *NGAL* neutrophil gelatinase-associated lipocalin, *SCr* serum creatinine

**Figure S2.** Summary of total reference numbers, disease distribution and sample type analysis of the miRNA candidates.


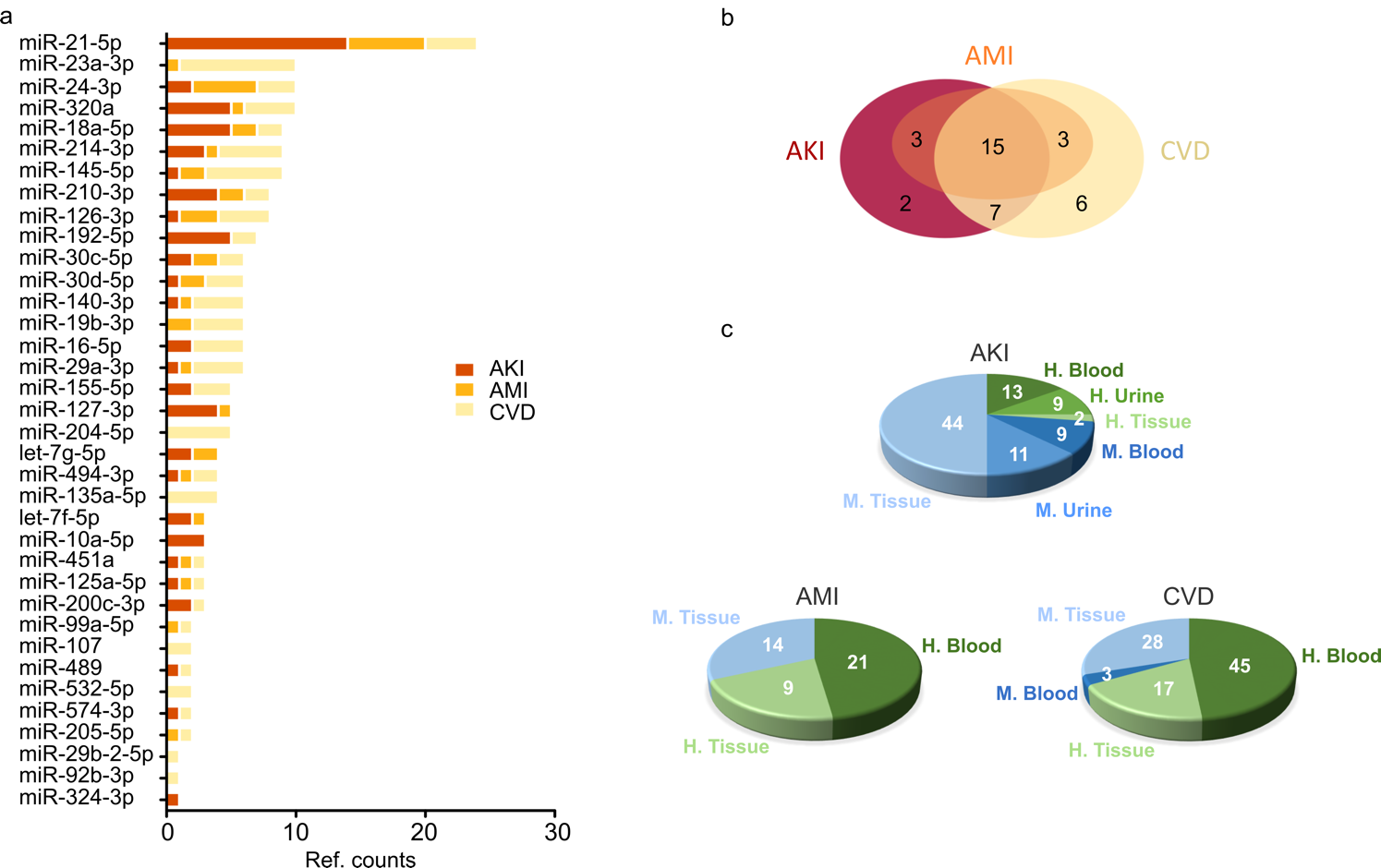


**a** Number of references related to the three disease types (AKI, AMI and non-AMI CVD) are listed for each miRNA. **b** Venn diagram summarizing the numbers of miRNAs associated with AKI, AMI and CVD. **c** Pie charts showing the distribution of the sample types and species investigated in the reports reviewed for AKI, AMI and CVD. *AKI* acute kidney injury, *AMI* acute myocardial infarction, *CVD* cardiovascular diseases, *H*, human, *M*, murine

Table S3 Kruskal-Wallis test and post-hoc Dunn’s multiple comparisons of the 17 miRNAs among all groups (N=108)

|  | miRNA level | Kruskal-Wallis test | Dunn’s multiple comparisons | | |
| --- | --- | --- | --- | --- | --- |
| miRNA | mean (n = 108) | *P*-value | AMI^+^AKI^-^ vs. AMI^-^AKI^-^ | AMI^-^AKI^+^ vs. AMI^-^AKI^-^ | AMI^+^AKI^+^ vs. AMI^+^AKI^-^ |
|  |  |  |  |  |  |
| miR-24 | 12.11 | 0.000 | 0.007 | 0.082 | 0.001 |
| miR-23a | 10.96 | 0.000 | 0.022 | 0.266 | 0.003 |
| miR-126 | 9.77 | 0.023 | 0.595 | 1.000 | 0.105 |
| miR-574 | 8.72 | 0.022 | 0.139 | 1.000 | 0.116 |
| miR-145 | 8.53 | 0.000 | 0.040 | 0.945 | 0.012 |
| miR-21 | 7.25 | 0.001 | 1.000 | 0.020 | 0.151 |
| miR-30c | 7.16 | 0.000 | 0.090 | 0.356 | 0.004 |
| let-7f | 6.34 | 0.001 | 0.234 | 1.000 | 0.002 |
| let-7g | 6.25 | 0.000 | 0.172 | 0.312 | 0.002 |
| miR-214 | 6.19 | 0.014 | 0.051 | 1.000 | 1.000 |
| miR-18a | 5.08 | 0.000 | 0.107 | 0.394 | 0.001 |
| miR-125a | 5.07 | 0.001 | 0.106 | 1.000 | 0.001 |
| miR-127 | 4.37 | 0.039 | 0.134 | 0.066 | 1.000 |
| miR-10a | 4.25 | 0.041 | 1.000 | 1.000 | 0.041 |
| miR-494 | 2.49 | 0.321 | - | - | - |
| miR-204 | 1.90 | 0.204 | - | - | - |
| miR-155 | 0.95 | 0.001 | 0.003 | 1.000 | 0.130 |

*AMI* acute myocardial infarction, *AKI* acute kidney injury
